# Do Performance-Based Measures and Behavioral Ratings of Executive Functioning Complement Each Other in Predicting Reading and Mathematics in Chinese?

**DOI:** 10.3390/bs13100823

**Published:** 2023-10-06

**Authors:** George K. Georgiou, Li Zhang

**Affiliations:** 1 Faculty of Education, University of Alberta, Edmonton, AB T6G 2G5, Canada; 2 Chengdu Shishi Tianfu High School, Chengdu 610042, China; zhangli_eleen@126.com

**Keywords:** Chinese, executive functioning, mathematics, reading, working memory

## Abstract

We examined what executive functioning (EF) components predict reading and mathematics within the same study and whether the effects of behavioral ratings of EF overlap or complement those of performance-based measures. One hundred and nine Grade 2 Mandarin-speaking Chinese students from Chengdu, China (55 girls, 54 boys, Mage = 8.15 years), were assessed on measures of EF (planning, inhibition, shifting, and working memory), speed of processing, reading and mathematics. Parents also rated their children’s EF skills using the Childhood Executive Functioning Inventory. Results of hierarchical regression analyses showed that only working memory among the performance-based EF measures predicted reading and mathematics. In addition, none of the behavioral ratings of EF made a significant contribution to reading and mathematics after controlling for mother’s education and speed of processing. Taken together, these findings suggest that working memory is a domain general predictor of academic achievement, but only when measured with cognitive tasks.

## 1. Introduction

Executive functioning (EF), broadly defined as a set of abilities that an individual uses in order to achieve a goal [1], has been shown to be a significant predictor of academic achievement (e.g., [2,3,4,5,6,7]). Although different conceptualizations of EF have been proposed in the literature (see [8], for a review), researchers concur that the following four are the most popular EF components: (1) planning, the ability of an individual to develop strategies to solve a problem, monitor the progress, and revise accordingly; (2) inhibition, the ability of an individual to suppress prepotent responses when necessary; (3) shifting (often called cognitive flexibility), the ability of an individual to switch between strategies or tasks; and (4) working memory, the ability to maintain information in short-term memory while processing other information. Meta-analytic studies estimated the average correlation between these EF components and academic achievement to range from 0.21 to 0.35 (see [9,10,11,12]). Despite evidence on the importance of the EF components in academic achievement, it remains unclear if the components of EF that predict reading also predict mathematics when included in the same study and whether different ways of operationalizing EF (performance-based vs. behavioral ratings) complement each other in predicting academic achievement. Thus, the purpose of this study was to examine what components of EF (measured with performance-based cognitive tasks and parent ratings) predict reading and mathematics performance in a sample of Chinese children.

### 1.1. The Relation of EF Components with Academic Achievement

There are good theoretical reasons why different components of EF may predict aca- demic achievement (e.g., [13,14,15,16,17]). Planning, the pinnacle of EF, is important in reading comprehension and problem solving as both academic outcomes require the selection, implementation, and adaptation of strategies when answering questions or solving problems [13]. Likewise, because working memory allows children to hold previously read information in their memory while simultaneously accessing new information, it is important for reading comprehension and problem solving that involves multiple steps in reaching a solution [15,18]. In regard to inhibition, researchers have argued that during word reading, children must inhibit activation of similarly looking words in long-term memory in order to accurately read a specific word. In addition, during reading comprehension, children must focus on information that is relevant to the main topic and ignore other that is often included in the text [15]. Similar to reading, in mathematics, children must suppress competing responses when retrieving arithmetic facts from memory [16,19]. Finally, shifting may help children to flexibly switch between the different ideas presented in a text or between one type of procedure or arithmetic operation to another [15,16,19].

To our knowledge, only a handful of studies have examined the role of different EF components in both reading and mathematics within the same study and have reported mixed findings (see [2,4,14,20,21]). On the one hand, Morgan et al. [16] and Nguyen and Duncan [17] found that all EF components in kindergarten were unique predictors of reading and mathematics in Grade 2. In turn, Cantin et al. [4] found that whereas inhibition, shifting and working memory were unique predictors of reading comprehension, only shifting was a unique predictor of mathematics. In a cross-cultural study with Grade 1 Canadian and Chinese children, Georgiou et al. [2] found that only working memory was a significant predictor of both reading and mathematics in the Canadian sample and that inhibition and working memory were significant predictors of reading and mathematics in the Chinese sample. Only Gerst et al. [14] included planning in their analyses and showed that the teacher ratings of planning (but not the cognitive measure of planning, i.e., Tower of London) was a significant predictor of reading comprehension and that neither planning task predicted math calculations.

### 1.2. Measurement of EF

A topic that has received much less attention by researchers is whether performance-based assessments and behavioral ratings of EF (completed either by parents or teachers) produce the same results when used to predict academic achievement within the same study. Understanding to what extent different measures of EF share variance or exert independent effects on academic achievement could verify their validity and also provide us with a more refined view of the role of EF components in academic achievement. What is rather intriguing in this line of research is that these two ways of obtaining information about children’s EF skills correlate only weakly with each other (e.g., [14,22,23], see also [24] for a meta-analysis). For example, Gerst et al. [14] showed that among the teacher-rated EF subscales, only inhibition and shifting correlated significantly with their cognitive counterpart, with the respective correlation being 0.25. The correlations for the working memory and planning tasks were non-significant. Given that behavioral rating scales are often used in clinical settings to identify children with executive dysfunction (probably because of the easiness of collecting this kind of data), one would expect that they produce similar results to performance-based measures of EF. Arguably, if the performance-based measures of EF and the behavioral ratings correlate only weakly with each other, then they do not likely measure the same skill. In fact, Toplak et al. [24] concluded that the different ways of measuring EF capture different information: performance-based measures represent efficiency of performance in an optimal and highly structured setting (e.g., laboratory), whereas behavioral-rating scales represent the frequency of goal achievement in a more authentic environment (e.g., home). 

To our knowledge, only one of two studies have compared the predictive value of performance-based and behavioral ratings of EF in reading and mathematics (we acknowledge that two more studies have compared the contribution of performance-based measures and behavioral ratings of EF to academic outcomes, but they either focused on predicting reading alone [25] or mathematics and spelling [22]). More specifically, Gerst et al. [14] showed that both types of working memory were complementary in the prediction of reading comprehension and math calculations. In regard to shifting and inhibition, the teacher ratings did not add any unique variance to the prediction of math calculations beyond that accounted for by the performance-based measures. In contrast, when predicting reading comprehension, the performance-based measures of inhibition and shifting did not add any unique variance to the teacher ratings. Finally, only the teacher ratings of planning predicted reading comprehension, while neither type of planning assessment predicted math calculations. Ten Eycke and Dewey [26] also examined the role of different performance-based measures and parent ratings of EF in reading and mathematics in a heterogeneous group of 5- to 18-year-old children. Their results showed that the parent ratings of EF (BRIEF composite score) was a unique predictor of both reading and mathematics (WIAT-II composite score) over and above the effects of performance-based measures of EF. However, when they reran their analyses using only the different subscale scores of BRIEF as predictors of reading and mathematics performance, none of the subscale scores predicted reading and only shifting and emotional control predicted mathematics. Clearly more research is needed examining the unique and shared variance in predicting reading and mathematics performance between the different ways of measuring EF.

### 1.3. The Present Study

The primary goal of this study was to examine which EF components predict reading and mathematics performance in a sample of Chinese children. In addition, we aimed to examine if the two methods of measuring EF (performance-based and behavioral ratings) would make unique contributions to the prediction of reading and mathematics outcomes. Based on the finding of previous studies (e.g., [2,14,22]), we expected that both methods of measuring working memory would make a unique contribution to both reading and mathematics (particularly to reading comprehension and problem solving). We did not formulate any specific hypotheses for the rest of the EF components because the previous studies produced mixed findings. 

Because most of our performance-based measures of EF as well as reading and mathematics tasks were speeded, we also controlled for speed of processing prior to examining the contribution of EF components to reading and mathematics. This approach was necessary in order to capture the “true” effect of EF components on academic achievement that is not confounded by the speed factor [27,28].

## 2. Method

### 2.1. Participants

One hundred and nine Mandarin-speaking Grade 2 Chinese children (55 girls, 54 boys, *M*_age_ = 8.15 years, *SD* = 0.33) were recruited on a voluntary basis from a larger sample of 130 children to participate in this study. The children were attending two public elementary schools in Chengdu, China, and mostly came from upper-middle class families (based on parents’ education, see below). None of the children were diagnosed with any intellectual, behavioral or sensory difficulties. Parental consent was obtained prior to testing the children. In addition, all children gave their oral assent prior to participating in any testing. The study was conducted in accordance with the Declaration of Helsinki, and the protocol was approved by the Ethics Committee of the University of Alberta (Pro00027309).

The parents of the children also participated in the study by filling out the Childhood Executive Functioning Inventory (CHEXI, see below for more information) and by providing information on mother’s highest achieved educational level. One hundred and four of the CHEXI questionnaires were filled out by mothers, two by both parents, and three by grandparents (the grandparents indicated that the parents were working out of town during the period of the study and they were the ones taking care of the child). The mean mother’s education level was similar to that reported in previous studies in metropolitan cities like Beijing and Shanghai [29,30].

### 2.2. Measures

#### 2.2.1. Speed of Processing

To assess speed of processing we administered the Visual Matching task [31]. Children were presented with 60 rows of numbers (e.g., 25, 38, 25, 59, 21, 73) and were asked to circle the two identical numbers in each row within a 3 min time limit. The first 20 rows included single-digit numbers, followed by 20 rows of two-digit numbers, and 20 rows of three-digit numbers. A participant’s score was the total number of correctly completed rows within the time limit. Cronbach’s alpha reliability in our sample was 0.82.

#### 2.2.2. Childhood Executive Functioning Inventory (CHEXI) 

CHEXI was developed by Thorell and Nyberg [32] and was adapted in Chinese by Thorell et al. [33]. Parents were asked to rate their children on 24 statements (e.g., When asked to do several things, they only remember the first or last) using a Likert scale that ranged from 1 (definitely not true) to 5 (definitely true). The 24 statements are used to form the following two constructs: working memory (13 items) and inhibition (11 items). Cronbach’s alpha reliability in our sample was 0.80 for working memory and 0.82 for inhibition.

#### 2.2.3. Planning

To assess planning, we administered the Planned Codes task [34]. Children were asked to fill in as many empty boxes as possible with a combination of Os and Xs that corresponded to a letter (e.g., A = OX, B = XX, C = OO, D = XO) that was printed at the top of each empty box. The task contained two pages, each with a distinct set of codes. At the top of each page the children could see the combination of Os and Xs that corresponded to each letter. The participants were allowed 1 min to fill in as many empty boxes as possible and they were told that they could use whatever strategy they wanted to reach their goal. A child’s score was the sum of correctly completed boxes across the two pages. Cronbach’s alpha reliability in our sample was 0.88.

#### 2.2.4. Inhibition/Shifting

To measure inhibition and shifting we administered the inhibition and switching task from NEPSY-II [35]. The inhibition task required children to say the opposite of the direction each arrow was pointing to (e.g., say up when the arrow is pointing down and say down when the arrow is pointing up). The arrows were arranged in an array consisting of five rows and eight columns. The time to name all stimuli in the card was the participant’s score. In shifting, children were asked to say the arrow’s correct direction if the arrow was colored black and say the arrow’s opposite direction if the arrow was white. The time to name all stimuli in the card was the participant’s score. A higher score in both inhibition and shifting tasks indicated poorer performance. Cronbach’s alpha reliability in our sample was 0.88 for inhibition and 0.82 for shifting.

#### 2.2.5. Working Memory

To assess working memory, we administered the Backward Digit Span task from the Wechsler Intelligence Scale for Children-III [36]. Children were asked to first listen carefully to the examiner saying out loud a string of digits and then repeat the sequence of digits in the reverse order. The string of digits started with only two digits and one digit was added at each difficulty level (the maximum length was eight digits). The task was discontinued after the child failed both trials of a given length. A child’s score was the maximum length of the digit string recalled correctly. Cronbach’s alpha reliability in our sample was 0.84.

#### 2.2.6. Reading

To assess reading, we used the following two measures: Sentence Verification and Passage Comprehension. Sentence Verification is a measure of reading fluency that was adopted from Pan et al. [37] and has been used in several previous studies in Chinese (e.g., [38,39]). The task required children to silently read sentences as quickly as possible and judge the truthfulness of each sentence by writing an √ or an X at the end of each sentence (e.g., The sun rises in the west…). The task consisted of 100 sentences that were arranged from short to long across the test. A child’s score was the total number of correct answers minus the number of incorrect within a 3 min time limit. Cronbach’s alpha reliability in our sample was 0.85. In turn, Passage Comprehension [40] was used to assess reading comprehension. Children were asked to read a narrative passage and then answer 18 multiple-choice questions. The title of the passage was “Prince Nezha Conquers the Dragon King” (selected from The Journey to the West by Wu Chengen). Each multiple-choice question had four options. Children were given 10 min to complete the task. A participant’s score was the total number of correct answers (max = 18). Cronbach’s alpha in our sample was 0.90.

#### 2.2.7. Mathematics

To assess mathematics, we administered the following two tasks: the Basic Arithmetic Test (BAT, [41]) and the Word Problems task [42]. BAT was used to assess calculation fluency. Children were asked to answer as many calculation problems as possible within a 3 min time limit. The task consisted of 28 problems, 14 additions (e.g., 2 + 1 = ? and 3 + 4 + 6 = ?) and 14 subtractions (e.g., 4 − 1 = ? and 20 − 2 − 4 = ?), that were mixed up and presented over two pages. The score was the total number of correct answers. Cronbach’s alpha reliability in our sample was 0.90. In turn, the Word Problems task, from the NMART test array [42], was used to assess problem solving. This task consisted of 20 problems that covered all four arithmetic operations—addition, subtraction, multiplication, and division as well as their combinations (e.g., A book costs 12 Yuan, and a comic book costs 15 Yuan. Guo bought two books and five comic books. How much would he get back from 100 Yuan?). One point was given for each correct answer and a child’s score was the total number of correct answers (max = 20) within a 5 min time limit. Cronbach’s alpha reliability in our sample was 0.94.

### 2.3. Procedure

Testing was completed in two sessions. In Session 1, children were individually tested on the planning, working memory, speed of processing and inhibition/shifting tasks in a quiet space in their school by a trained graduate student. Testing lasted approximately 30 min. Session 2 included the reading and mathematics tasks and was completed in the children’s classroom as a whole group activity. Session 2 lasted approximately 30 min and was conducted 10 days after Session 1 began. Finally, CHEXI took 5–7 min to complete and was filled out by the parents during the same time as their children’s testing in Session 1.

### 2.4. Statistical Analysis

First, we calculated the descriptive statistics of our measures (means, SDs, max and min values) and examined if there were any violations of normality by inspecting the Q-Q plots and the Shapiro–Wilk tests. Second, we calculated the Pearson product moment correlations between our measures. Finally, to examine what EF components predict reading and mathematics skills we performed hierarchical regression analyses. First, we entered the mother’s education at Step 1 of the regression equation as a control variable. Next, we entered speed of processing at Step 2. Finally, at Step 3, we entered either the two CHEXI subscales or the four performance-based measures of EF (planning, working memory, inhibition and shifting) as a block. Although we were also planning to perform hierarchical regression analyses with the pairs of EF tasks that would be unique predictors of reading or mathematics in the above set of hierarchical regression analyses, none of the CHEXI subscales made a significant contribution to reading and mathematics outcomes (see Results Section), and for this reason we did not run this analysis.

## 3. Results

Table 1 presents the descriptive statistics of our measures. Before conducting any further analyses, we examined the distributional properties of our measures. The scores of a few outliers (one at the high end of the Sentence Verification distribution, three at the low end of the Word Problems distribution and two at the high end of the inhibition distribution) were winsorized to the next non-outlier’s score plus or minus one. An examination of the Q-Q plots and Shapiro–Wilk tests indicated no significant deviations from normality. Next, we calculated the Pearson product moment correlations between our measures (see Table 2). Irrespective of the way EF was measured, the correlations of the EF measures with the reading and mathematics tasks were relatively low. The highest correlation was between inhibition-NEPSY and BAT (*r* = −0.34). With one exception (Digit Span Backward with working memory-CHEXI), the performance-based measures of EF did not correlate significantly with the EF scores from CHEXI.

Next, we performed hierarchical regression analyses to examine the role of the EF components in reading and mathematics. Prior to conducting these analyses, we also checked if the assumptions of multiple regression analyses were met. First, our dependent variables were normally distributed. Second, we did not have multicollinearity (both the tolerance values and the VIF values were within the recommended range). Finally, homoscedasticity and linearity were checked by visually inspecting the residual plots. Again, all values were within the acceptable range.

Table 3 presents the results of the hierarchical regression analysis with the reading outcomes, and Table 4 presents the results with the mathematics outcomes. Standardized beta coefficients from the step in which the variables were entered into the regression equation along with R2 changes associated with each step are presented at each table. The results of Table 3 show that after controlling for mother’s education and speed of processing, only the performance-based measure of working memory made a significant contribution to Sentence Verification (β = 0.176, *p* < 0.05) and Passage Comprehension (β = 0.176, *p* < 0.05). None of the CHEXI scores of EF made a significant contribution. The results in Table 4 with the mathematics outcomes were similar to those with the reading outcomes. The only EF component that survived the statistical control of mother’s education and speed of processing was the performance-based measure of working memory and only when predicting word problems (β = 0.175, *p* < 0.05). It should be noted here that if we had not controlled for speed of processing, planning would also predict Sentence Verification (β = 0.214, *p* < 0.05) and inhibition-NEPSY would also predict BAT (β = −0.283, *p* < 0.05). 

## 4. Discussion

The objective of this study was to examine which EF components were predictive of reading and mathematics outcomes and if there was a difference between two ways of measuring EF (behavioral-based vs. parent ratings). Our results showed that after controlling for mother’s education and speed of processing, only performance-based working memory was a unique predictor of both reading outcomes and problem solving. The unique contribution of working memory in reading comprehension and problem solving was not surprising given that children should retain important information about the passage they read or the problem they must solve in order to answer questions about the passage or solve the problem. Previous studies have also shown working memory to be a significant predictor of reading comprehension and problem solving (e.g., [2,14,16,24]). The fact that working memory also predicted reading fluency in our study is likely due to the task used to measure reading fluency (i.e., Sentence Verification). In order to evaluate the truthfulness of each sentence, the children should retain the information provided in the sentence in their memory and then make a decision. Previous studies in which a word reading fluency task was used to operationalize reading fluency showed no significant effects of working memory (e.g., [43,44]).

To our surprise, none of the other performance-based EF components made a unique contribution to reading or mathematics despite the fact that some of them correlated significantly with the reading and mathematics outcomes (see Table 2). This is likely due to the inclusion of speed of processing as a control variable in the regression equation. Our decision to include speed of processing was intentional because some of the tasks used to measure EF (e.g., inhibition, planning) as well as some of the outcome measures (e.g., Sentence Verification, BAT) are speeded and the speed component of the tasks may inflate their relation. Indeed, when we removed speed of processing from the regression equation, planning predicted Sentence Verification and inhibition predicted BAT. The “impurity” of the EF tasks has been called out before by Miyake and colleagues [45], and other researchers have also suggested that speed of processing should be controlled prior to examining the contribution of EF components to reading or mathematics (e.g., [2,46,47]).

The second goal of this study was to examine if similar results could be obtained had we used parent ratings of children’s EF. Unfortunately, none of the parent-rated EF components (as measured with CHEXI) made a unique contribution to reading and mathematics after controlling for mother’s education and speed of processing. Before commenting on this finding, we should also note the non-significant relations of these measures with the behavioral-based EF components. With the exception of working memory, the parent-rated EF components did not correlate significantly with their children’s behavioral-based EF counterpart. This replicates the findings of previous studies (e.g., [14,18,48,49]) and suggests that we cannot use scores of EF for either predictive or diagnostic purposes interchangeably. Clearly, these two ways of obtaining information about children’s EF skills do not measure the same skill. Regarding the non-significant contribution of the parent-rated EF components to reading and mathematics, a possible explanation beyond the inclusion of speed of processing as a control variable is the questionnaire we used in our study. The questionnaire includes only 24 statements, and each of the EF components is operationalized with a relatively small number of statements. This may have inadvertently reduced our chances of finding significant effects. An alternative explanation may be that CHEXI is the only non-performance task used here. The results may have been different if we had used linguistic and mathematical tests of the frequency of goal achievement in a more authentic environment like home. Finally, questionnaires asking parents about their children are subject to a social desirability bias (e.g., [50,51]). This means that parents often respond with what they think the society would like to hear than what really happens at their home. This, in turn, may result in non-significant associations between the scores derived from these questionnaires and their children’s academic achievement. Certainly, our findings call for more research on the predictive value of CHEXI and suggest that we should perhaps look at alternative ways of garnering information about children’s EF skills in more authentic environments (see [52]).

Some limitations of the present study should be reported. First, this is a concurrent study and any significant relations between the measures do not imply causation. Second, as mentioned above, CHEXI is brief, and this may have prevented us from adequately capturing different aspects of children’s EF skills. We chose CHEXI because it was already available in Chinese [33] and parents would more likely fill out a 24-item questionnaire than an 86-item questionnaire (see BRIEF, [53]); furthermore, it had been used in previous studies examining the role of behavioral ratings of EF in children’s academic achievement [14,18,26]. Third, our participants were second graders, and our findings may not generalize to other grade levels. We mention this because the structure of EF as well as the relation of the EF components with academic achievement may change over time (see [54,55]). Fourth, we only contrasted performance-based measures of EF with behavioral ratings. Another way of obtaining data on EF is through direct observations (e.g., [52]). Unfortunately, not only our small grant was not able to cover the cost of such data collection, but also children in Chengdu are released from school after 6 pm and it would be impossible to obtain parental consent to observe them at home after having dinner and finishing their homework. Fifth, working memory is a multicomponential construct [56], and Digit Span Backward (the measure we used in our study) only measures the central executive component. Thus, we cannot make any arguments concerning the contribution of the phonological loop or visuospatial sketchpad. Finally, the performance-based EF components were operationalized with a single task. Unfortunately, we were only given 40 min by the school authorities to individually assess each child, and this did not allow us to administer more measures. Even though we used what researchers would consider “standard” measures of EF, a future study should replicate our findings with more measures of each construct. The use of a single measure may have particularly influenced planning, since previous studies (e.g., [57,58]) have shown that planning has different levels (i.e., operation planning, action planning) and Planned Codes (a measure of operation planning) may not be a strong predictor of higher-level comprehension tasks like the one used in our study.

To conclude, our findings add to those of previous studies that examined the role of different cognitive–linguistic skills in reading and mathematics (e.g., [2,59,60,61]) in an attempt to identify domain general and domain specific effects of these skills on academic achievement. After controlling for mother’s education and speed of processing, only performance-based working memory made a unique contribution to both reading outcomes and problem solving. This suggests that not all components of EF are important in academic achievement, particularly when the speed component that is shared between some of the performance-based EF tasks and the outcome measures is controlled for.

## Figures and Tables

**Table 1:** Descriptive statistics for the measures used in this study.

	M	SD	Min	Max	Skewness	Kurtosis
Sentence Verification	39.88	12.03	20	71	0.663	−0.043
Passage Comprehension	9.67	3.62	2	18	0.153	−0.613
BAT	23.97	2.91	16	28	−0.746	0.138
Word Problems	9.92	2.00	3	17	−0.563	1.606
Performance-based EF						
Inhibition ^a^	34.52	7.08	20.41	61.50	0.224	−0.469
Working memory	6.45	2.39	2	13	0.747	0.349
Shifting ^a^	46.47	7.75	27.50	68.17	0.239	−0.200
Planning	68.16	15.45	42	109	0.589	−0.085
Parent ratings of EF						
Inhibition	33.81	5.79	18	44	−0.262	−0.312
Working memory	33.09	6.97	16	50	0.069	0.097
Speed of processing	35.14	5.19	25	46	0.033	−0.819
Mother’s education ^b^	5.87	0.77	3	7	−0.515	0.204

Note: BAT = Basic Arithmetic Task; ^a^ measured in seconds; ^b^ mother’s education included seven categories as follows: (a) completed Grade 3 or a lower grade level, (b) completed Grade 4 to 6, (c) completed junior high school, (d) completed senior high school, (e) graduated from a college, (f) graduated from a university, and (g) completed graduate studies (e.g., master’s or PhD).

**Table 2:** Correlations between our measures.

		1.	2.	3.	4.	5.	6.	7.	8.	9.	10.	11.	12.
1.	Mother’s education		0.19 *	−0.06	0.11	0.01	−0.06	−0.26 **	−0.17	0.05	0.03	0.19 *	0.17
2.	Visual Matching			0.22 *	0.22 *	−0.25 **	−0.31 *	−0.24 *	−0.23 *	0.42 **	0.29 **	0.50 **	0.52 **
3.	Planned Codes				0.12	−0.27 **	−0.31 **	0.05	−0.02	0.27 **	0.16	0.15	0.14
4.	DSB					−0.14	−0.10	−0.16	−0.20 *	0.28 **	0.29 **	0.17	0.30 **
5.	Inhibition-NEPSY						0.64 **	0.08	0.07	−0.21 *	−0.09	−0.34 **	−0.22 *
6.	Shifting-NEPSY							0.18	0.14	−0.12	0.02	−0.25 **	−0.24 *
7.	Inhibition-CHEXI								0.68 **	−0.14	−0.06	−0.16	−0.21 *
8.	WM-CHEXI									−0.19 *	−0.16	−0.11	−0.24 *
9.	Sent. Verification										0.66 **	0.44 **	0.51 **
10.	Passage Comp.											0.39 **	0.48 **
11.	BAT												0.55 **
12.	Word Problems												

Note: DSB = Digit Span Backward; WM = working memory; BAT = Basic Arithmetic Test. * *p* < 0.05, ** *p* < 0.01.

**Table 3:** Results of the hierarchical regression analysis predicting reading skills.

		Sentence Verification		Passage Comprehension	
Step	Variables	β	ΔR^2^	β	ΔR^2^
1.	Mother’s Education	0.052	0.00	0.048	0.00
2.	Speed of Processing	0.440 ***	0.18 ***	0.296 **	0.08 **
3.	Planning	0.171	0.07 *	0.100	0.08 *
	Inhibition	−0.131		−0.084	
	Shifting	0.134		0.171	
	WM	0.176 *		0.235 *	
3.	Inhibition-CHEXI	0.049	0.01	0.161	0.02
	WM-CHEXI	−0.145		−0.219	

Note: WM = working memory; * *p* < 0.05, ** *p* < 0.01, *** *p* < 0.001.

**Table 4:** Results of the hierarchical regression analysis predicting mathematics skills.

		BAT		Word Problems	
Step	Variables	β	ΔR^2^	β	ΔR^2^
1.	Mother’s Education	0.201 *	0.04 *	0.165	0.03
2.	Speed of Processing	0.497 ***	0.23 ***	0.503 ***	0.25 ***
3.	Planning	0.001	0.04	−0.005	0.04*
	Inhibition	−0.219		−0.010	
	Shifting	0.027		−0.096	
	WM	0.028		0.175 *	
3.	Inhibition-CHEXI	−0.065	0.00	0.003	0.01
	WM-CHEXI	0.067		−0.118	

Note: WM = working memory; * *p* < 0.05, *** *p* < 0.001.

## Data Availability

The data can be shared upon request from the corresponding author.
